# D-dopachrome tautomerase activates COX2/PGE_2_ pathway of astrocytes to mediate inflammation following spinal cord injury

**DOI:** 10.1186/s12974-021-02186-z

**Published:** 2021-06-11

**Authors:** Huiyuan Ji, Yuxin Zhang, Chen Chen, Hui Li, Bingqiang He, Ting Yang, Chunshuai Sun, Huifei Hao, Xingyuan Zhang, Yingjie Wang, Yue Zhou, Zhenjie Zhu, Yuming Hu, Aihong Li, Aisong Guo, Yongjun Wang

**Affiliations:** 1 Key Laboratory of Neuroregeneration of Jiangsu and Ministry of Education, Co-innovation Center of Neuroregeneration, Nantong University, Nantong, 226001 People’s Republic of China; 2 Department of Rehabilitation Medicine, Affiliated Hospital of Nantong University, Nantong, 226001 People’s Republic of China; 3 Department of Rehabilitation Medicine, Shanghai Ninth People’s Hospital Affiliated to Shanghai Jiao Tong University School of Medicine, Huangpu District, Shanghai, 200011 People’s Republic of China; 4 Department of Neurology, Affiliated Hospital of Nantong University, Nantong, 226001 People’s Republic of China

**Keywords:** D-DT, MIF, Spinal cord, Astrocyte, Inflammation, PGE_2_, Injury, Central nervous system, COX2, CD74

## Abstract

**Background:**

Astrocytes are the predominant glial cell type in the central nervous system (CNS) that can secrete various cytokines and chemokines mediating neuropathology in response to danger signals. D-dopachrome tautomerase (D-DT), a newly described cytokine and a close homolog of macrophage migration inhibitory factor (MIF) protein, has been revealed to share an overlapping function with MIF in some ways. However, its cellular distribution pattern and mediated astrocyte neuropathological function in the CNS remain unclear.

**Methods:**

A contusion model of the rat spinal cord was established. The protein levels of D-DT and PGE_2_ synthesis-related proteinase were assayed by Western blot and immunohistochemistry. Primary astrocytes were stimulated by different concentrations of D-DT in the presence or absence of various inhibitors to examine relevant signal pathways. The post-injury locomotor functions were assessed using the Basso, Beattie, and Bresnahan (BBB) locomotor scale.

**Results:**

D-DT was inducibly expressed within astrocytes and neurons, rather than in microglia following spinal cord contusion. D-DT was able to activate the COX2/PGE_2_ signal pathway of astrocytes through CD74 receptor, and the intracellular activation of mitogen-activated protein kinases (MAPKs) was involved in the regulation of D-DT action. The selective inhibitor of D-DT was efficient in attenuating D-DT-induced astrocyte production of PGE_2_ following spinal cord injury, which contributed to the improvement of locomotor functions.

**Conclusion:**

Collectively, these data reveal a novel inflammatory activator of astrocytes following spinal cord injury, which might be beneficial for the development of anti-inflammation drug in neuropathological CNS.

**Supplementary Information:**

The online version contains supplementary material available at 10.1186/s12974-021-02186-z.

## Background

Astrocytes are the predominant glial cell type in the central nervous system (CNS) performing a wide array of neurophysiological functions [1, 2]. It is now clear they are essential for neuronal synaptogenesis, blood–brain barrier (BBB) formation, ion and water homeostasis, as well as recycle of neurotransmitter [3, 4]. Astrocytes are able to respond to and transmit danger signals *via* conversion of phenotype and secretion of cytokines and chemokines [5, 6]. Similar to that of the microglia, the membrane of astrocytes expresses a wide array of pattern recognition receptors (PRRs), which are able to interact with damage-associated molecular patterns (DAMPs) to trigger inflammatory responses [7]. Therefore, astrocytes are recognized as an important contributor to inflammation-related neuropathology in CNS [8]. In fact, astroglial inflammation not only correlates with the progression of several CNS degenerative diseases, but also deteriorates microenvironment of injured CNS unfavorable to nerve regeneration [1, 9, 10]. A selective inhibition of astrocyte-activated inflammation by reducing NFκB activity has been shown to result in protective effects on the axon and functional recovery following spinal cord injury (SCI) [9, 11]. Taken together, astrocytes are active players in the innate immunity of CNS, thereby has drawn much attention when dealing with CNS inflammation.

Prostaglandin E_2_ (PGE_2_) is commonly considered to be a potent proinflammatory mediator, though in some contexts show the converse effects [12, 13]. It is also the final mediator of fever, which initiates thermogenesis by binding to its EP3 receptor subtype in the preoptic hypothalamus [14]. This lipid mediator is derived from arachidonic acid (AA) metabolism *via* the activation of the cyclooxygenase (COX) pathway. In the CNS, at least two COX isoforms, the constitutive (COX1) and inducible (COX2) isoforms, are expressed in neuronal and glial cells responsible for the production of the PGE_2_ [15–17]. Both astrocytes and microglia are considered to be major sources of PGE_2_ and other prostanoids within the CNS after injury or neurological disorders [17–19]. The DAMPs released by necrotic cells or secreted by inflammatory cells are able to activate the COX2/PGE_2_ signal pathway of astrocytes, which in turn promotes various types of CNS damages, such as multiple sclerosis, Alzheimer’s disease, Parkinson’s disease, and functional loss of the spinal cord [17, 20]. However, the associated cytokines/factors responsible for COX2 activation in astrocytes, as well as the underlying mechanisms are not fully elucidated.

The macrophage migration inhibitory factor (MIF) has been demonstrated to facilitate COX2 expression in multiple cell types through the regulation of mitogen-activated protein kinases (MAPKs) [17, 21–23]. As a potent proinflammatory cytokine, MIF can be produced by various cell types including monocytes, macrophages, T and B lymphocytes, and hepatocytes [24]. MIF is also inducibly expressed in the neurons, astrocytes, and microglia of the CNS to augment neuroinflammation and related neuropathology [25–27]. A closely homologous protein of MIF, D-dopachrome tautomerase (D-DT), has been described to show not only three-dimensional structural similarity, but also share receptors and biological functions with MIF [28–30]. D-DT protein is ubiquitously expressed in all tissues with the highest levels detected in the liver and testis. Inflammatory stimuli or hypoxia stress is sufficient to promote a rapid release of the protein from tumor, inflammatory, and damaged or necrotic cells [30, 31]. To date, less information regarding to physiopathological functions of D-DT is available, except for its cancer- and inflammation-related activities overlapping with those of MIF [28, 29]. A detailed analysis of D-DT functional motifs has revealed that D-DT lacks a pseudo (E)LR domain in association with mediating MIF’s binding with the non-canonical, chemokine receptor CXCR2 [32]. Also, the active site and the surrounding area of D-DT protein are differentially charged comparing to those of MIF [33–35]. These structural differences result in distinct biological activity between the two homologs. For example, D-DT binds the MIF receptor CD74 with high affinity, but has higher dissociation rate than MIF [30]. MIF positively, while D-DT negatively contributes to adipose tissue inflammation [36, 37]. Therefore, the differential regulatory mechanisms mediated by MIF and D-DT are expected in various cell types or tissues.

We have previously found that MIF participates in the activation of COX2/PGE_2_ signal pathway of astrocytes following SCI [17]. To ascertain whether the similar actions were taken by D-DT in the astrocytes, we analyzed the expression changes of D-DT, as well as the correlations with those of COX2 in the contused spinal cord. We further investigated the mechanism of D-DT-induced production of PGE_2_ in the astrocytes, and compared the effects of different inhibitors. Our results have provided a novel contributor of neuroinflammation following SCI, which might be a potential target for pharmacotherapy of CNS inflammation.

## Methods

### Animals

Adult male Sprague-Dawley (SD) rats, weighing 180–220 g, were provided by the Center of Experimental Animals, Nantong University. All animal experiments were approved by *the Animal Care and Use Committee of Nantong University* and the *Animal Care Ethics Committee of Jiangsu Province*. All rats were housed in standard cages (five rats in each cage) in an environment maintained at 22 ± 2 °C on a 12–12-h light–dark cycle and had free access to water and food.

### Establishment of contusion SCI rat model and drug treatment

The number of animals subjected to surgery was calculated by six per experimental group in triplicate. The contusion SCI rat model was prepared as the previous description [38]. In a nutshell, all animals were anesthetized with 10% chloral hydrate (3 mg/kg) administered intraperitoneally. The fur around the surgical site was shaved, and the skin was disinfected with iodophor. Then the spinous processes of T8–T10 vertebrae were surgically exposed, and a laminectomy was performed at the ninth thoracic vertebral level (T9) with the dura remaining intact. The exposed spinal cord segment (about 3 mm in length) received a 150-kilodyne contusion injury using the IH-0400 Impactor (Precision Systems and Instrumentation) injury device. The impact rod was removed immediately, and the wound was irrigated. For drug delivery, 8 μl of 100 mM MIF inhibitor 4-iodo-6-phenylpyrimidine (4-IPP; TOCRIS) or D-DT inhibitor 4-CPPC (AOBIOUS) were slowly injected intrathecally, prior to the incision suture. The rats were subcutaneously administered with 0.2 ml antibiotics following surgery. Manual expression of bladders was performed twice a day until animals recovered spontaneous voiding.

### Cell culture and treatment

Astrocytes were prepared from the spinal cord of newborn SD rats, 1-2 days after birth, and the astrocytes were isolated and cultured according to previously described methods [26]. Briefly, the spinal cords removed from the spinal canal were placed into 0.01 M PBS containing 1% penicillin–streptomycin. The spinal cord capsule was stripped clean under the microscope, followed by mincing with scissors, and digestion with 0.25% trypsin for 15 min at 37 °C. Digestion was terminated by addition of Dulbecco’s Modified Eagle’s Medium—high glucose medium containing 10% fetal bovine serum (FBS), 1% penicillin–streptomycin, and 1% L-glutamine. The suspension was then centrifuged at 1200 rpm for 5 min, and the cells were resuspended and seeded onto poly-L-lysine pre-coated culture flask in the presence of 5% CO_2_. The medium was changed every 3 days until the whole flask is covered with cells. After 7–9 days, the culture flask was shaken at 250 rpm overnight to remove non-astrocytes. Astrocyte phenotype was evaluated by cell exhibiting a characteristic morphology and positive staining for the astrocytic marker glial fibrillary acid protein (GFAP). Astrocytes with purity more than 95% are acceptable for subsequent experiments.

To determine the effects of the selective inhibitor NS398 (COX2), SB203580 (P38), SP600125, (JNK) or PD98059 (ERK) on the D-DT-induced astrocyte production of PGE_2_ and its synthesis-related proteins, the cells were treated with 1 μg/ml recombinant D-DT (Aviva Systems Biology) or MIF (ProSpec) in the presence or absence of 30 μM NS398 (Sigma), 10 μM SB203580 (TOCRIS), 10 μM SP600125 (TOCRIS) or 10 μM PD98059 (TOCRIS) for 24 h prior to assay.

For knockdown of CD74 expression in the astrocytes, the cells were cultured on the six-well plates for 24 h, followed by transfection of CD74 siRNA2 (sense strand 5'-CAG GAU AUG GGC CAA AUG U dTdT-3', antisense strand 5'-A CAU UUG GCC CAU AUC CUG dTdT-3') or scramble siRNA (sense strand 5'-GGC UCU AGA AAA GCC UAU GC dTdT-3', antisense strand 5'-GC AUA GGC UUU UCU AGA GCC dTdT-3') with iMAX transfection reagent (Invitrogen) for 24 h. The astrocytes were subsequently incubated at medium in the absence of 1% penicillin–streptomycin for 24 h, and then stimulated by 1 μg/ml D-DT recombinant protein for another 24 h before ELISA and Western blot assay.

### Western blot

Protein was harvested from cells with a buffer containing 50 mM Tris (pH 7.4), 150 mM NaCl, 1% Triton X-100, 1% sodium deoxycholate, 0.1% SDS, and 1 mM PMSF, following treatment with recombinant rat D-DT protein (Aviva Systems Biology) for 24 h. Alternatively, protein was extracted from 1-cm spinal segments of the injured site at 0 day, 1 day, 4 days, and 1 week following contusion (*n* = 6 in each time point). Samples were vortexed for 30 min and centrifuged at 12,000 rpm for 15 min. The supernatants were collected and stored at − 20 °C for use. Protein concentration of each specimen was measured by the BCA method to maintain the same loads according to the manufacturer’s instructions. Proteins were heated at 95 °C for 5 min, and 20 μg of each sample were electrophoretically separated on 10% SDS-PAGE gel, followed by transferring onto a polyvinylidene difluoride (PVDF) membrane. The membrane was blocked with 5% skim milk in Tris-buffered saline containing 0.1% Tween-20 for 1 h, and then an overnight incubation at 4 °C with primary antibodies: D-DT (1:500, Abcam); MIF (1:500, Abcam); COX1 (1:1000, CST); COX2 (1:1000, Cayman); mPGES-1 (1:200, Cayman); mPGES-2 (1:200, Cayman); cPGES (1:1000, Abcam); CD74 (1:1000, Biorbyt). After washing 3 times with TBST for 10 min each, the membrane was incubated with secondary antibody goat-anti-mouse HRP or goat-anti-rabbit HRP (1:1000, Beyotime) for 2 h at room temperature. The HRP activity was detected using an ECL kit. The image was scanned with a GS800 Densitometer Scanner (Bio-Rad), and the data were analyzed using PDQuest 7.2.0 software (Bio-Rad). β-actin (1:5000) was used as an internal control.

### ELISA

Cells or tissue samples were sonicated using the lysis buffer supplemented with a protease inhibitor PMSF as mentioned above. Homogenate was centrifuged at 12,000 rpm for 15 min at 4 °C, and the supernatant was collected for PGE_2_ ELISA assay (ARBOR ASSAYS) according to the manufacturer’s directions. The concentrations of PGE_2_ are expressed as picogram/milliliter (pg/ml). Plates were read with a multifunctional enzyme marker (Biotek Synergy2) at a 450-nm wavelength.

### Tissue immunofluorescence

The vertebra segments were harvested from six experimental models of each time point, post-fixed, and sectioned. The sections were blocked with 0.01 M PBS containing 3% BSA, 0.1% Triton X-100, and 10% normal goat serum for 1 h at 37 °C, and incubated overnight at 4 °C with primary antibodies: GFAP (1:400, Sigma); OX42 (1:200, Abcam); MBP (1:500, CST); NeuN (1:1000, Abcam); D-DT (1:200, Abcam); S100β (1:400, Abcam); COX2 (1:200, Cayman); CD74 (1:50, Bioss). Thereafter, the sections were rinsed with PBS and incubated with the Cy3-labeled goat anti-rabbit IgG (1:400, Proteintech) or the Alexa Fluor 488-labeled donkey anti-mouse IgG (1:400, Abcam). Sections were observed under a fluorescence microscope (ZAISS, axio image M2).

### Behavioral tests

The hindlimb locomotor function recovery was evaluated using the Basso, Beattie, and Bresnahan (BBB) locomotor scale as previously described (Zhou et al., 2018). Briefly, after intrathecal injection of 8 μl of 100 mM 4-CPPC or vehicle at 0, 7, 14, and 21 days, three well-trained investigators blind to the study were invited to observe the behavior of rats for 5 min. The BBB score ranged from 0 to 21 according to the rating scale. Every rat had a BBB score of 21 before surgery, and 0 to 1 after a successful SCI.

### Statistical analysis

Statistical analysis used GraphPad Prism 8 software (San Diego, CA, USA). Normality and homoscedasticity of the data were performed using Levene’s test. Independent sample t test and one-way analysis of variance (ANOVA) followed by Bonferroni’s post hoc comparisons tests were utilized for comparisons for different groups. All data were presented as mean ± standard deviation (M ± SD). Two-sided *p* value < 0.05 was considered statistically significant.

## Results

### Expression changes of D-DT protein and its regulatory correlations with COX2 activation following rat SCI

**Fig. 1:**
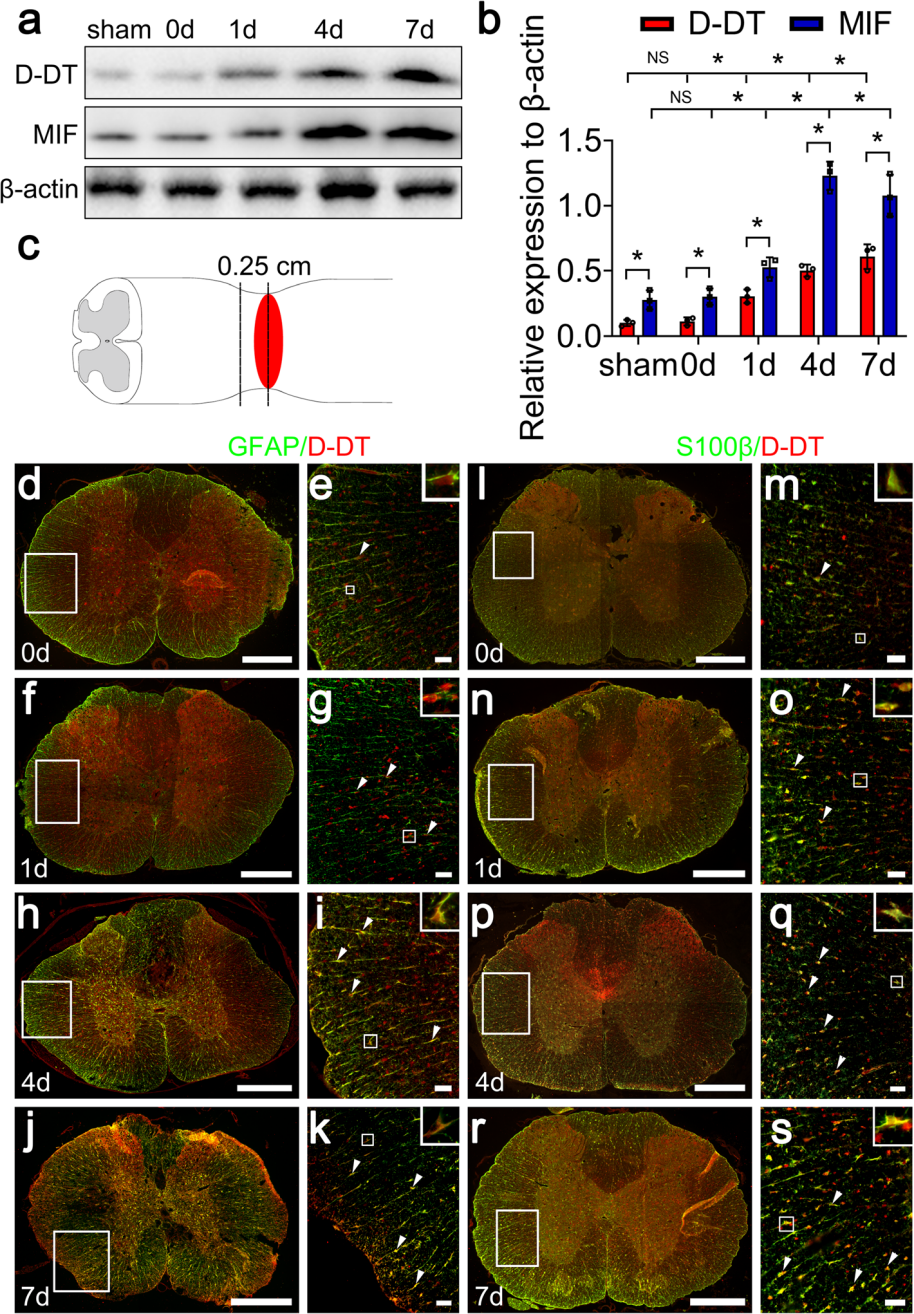
Examination of D-DT and MIF protein levels and colocalization with astrocytes following rat SCI. **a** Western blot analysis of D-DT and MIF following spinal cord injury at 0, 1, 4, and 7 days. The sham and 0-day group were used as control. **b** Quantification data as shown in (**a**). Quantities were normalized to endogenous β-actin. **c** Illustration of horizontal section sites at the contused cord. **d–s** Immunostaining showed colocalization of D-DT with GFAP- and S100β-positive astrocytes. Rectangle indicates region magnified. Arrowheads indicate the positive signals. Scale bars, 500 μm in (**d**), (**f**), (**h**), (**j**), (**l**), (**n**), (**p**) and (**r**); 50 μm in (**e**), (**g**), (**i**), (**k**), (**m**), (**o**), (**q**), and (**s**). Experiments were performed in triplicates. Error bars represent the standard deviation (*P < 0.05)

**Fig. 2:**
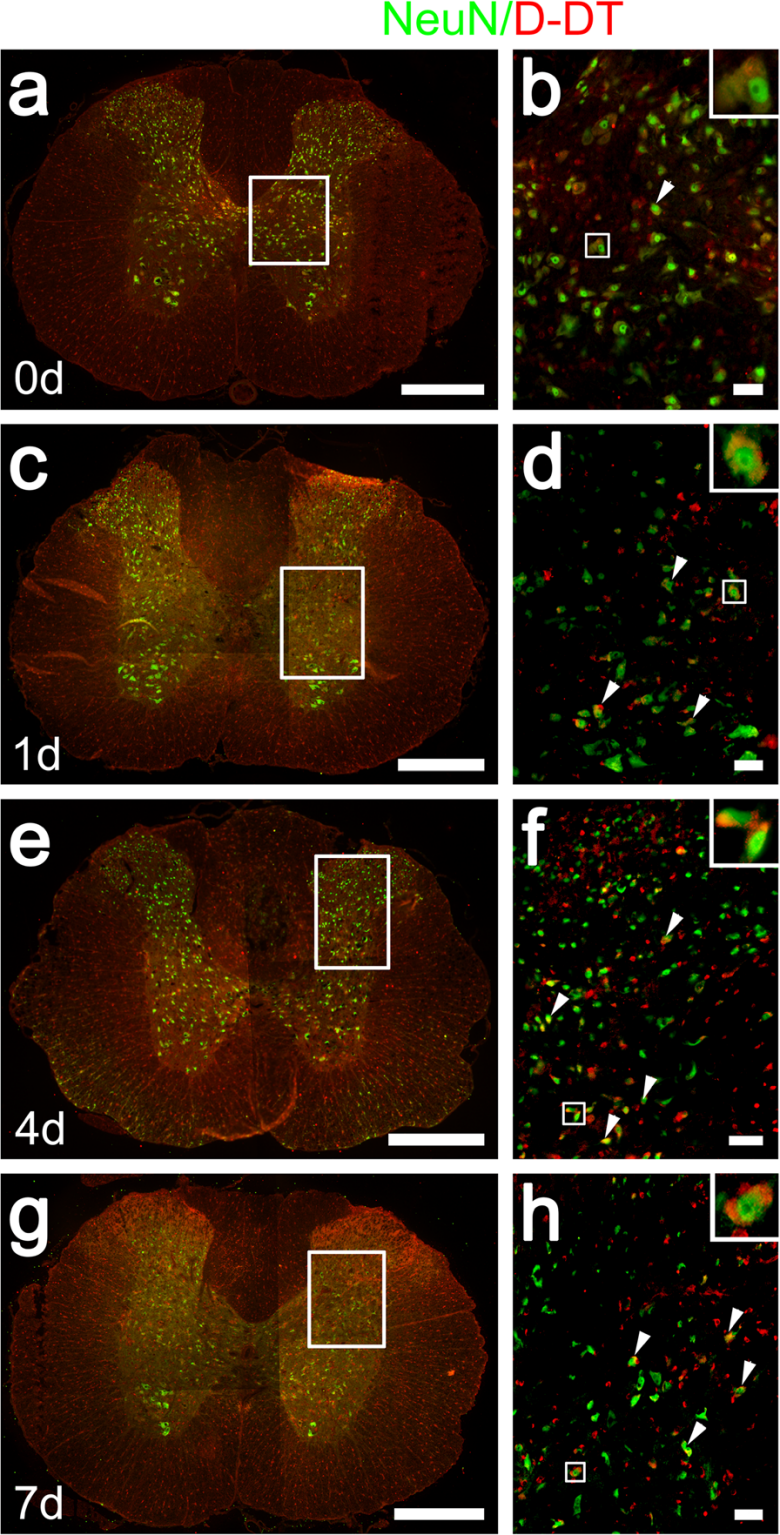
Analysis of D-DT distribution within neurons. **a–h** Immunostaining showed colocalization of D-DT with NeuN-positive neurons. Rectangle indicates region magnified. Arrowheads indicate the positive signals. Scale bars, 500 μm in (**a**), (**c**), (**e**), and (**g**); 50 μm in (**b**), (**d**), (**f**), and (**h**)

Although it is well established that D-DT is constitutively expressed in many detected tissues with most abundance in the liver, very few studies have examined its cellular distribution of the dopachrome tautomerase in the spinal cord, as well as its responses to the injury. Western blot was performed to quantify the protein levels of D-DT at 0 day, 1 day, 4 days, and 7 days following SCI. Meanwhile, its homologue MIF was also determined in parallel. Results demonstrated that the protein levels of D-DT and MIF significantly increased at lesion sites from 1 day onwards in comparison with the sham and 0-day control, indicating the similar injury-induced effect on the two homologues (Fig. 1a, b). Immunostaining was further carried out to observe the cell sources of D-DT protein. Cord sections were made from a 0.25-cm length to the epicenter of contusion (Fig. 1c). Results displayed that D-DT was colocalized with GFAP- and S100β-positive astrocytes (Fig. 1d–s), and NeuN-positive neurons (Fig. 2), rather than with OX42-positive microglia or MBP-positive oligodendrocytes (Additional file 1). The data indicate that D-DT is inducibly expressed within the astrocytes and neurons following SCI.

**Fig. 3:**
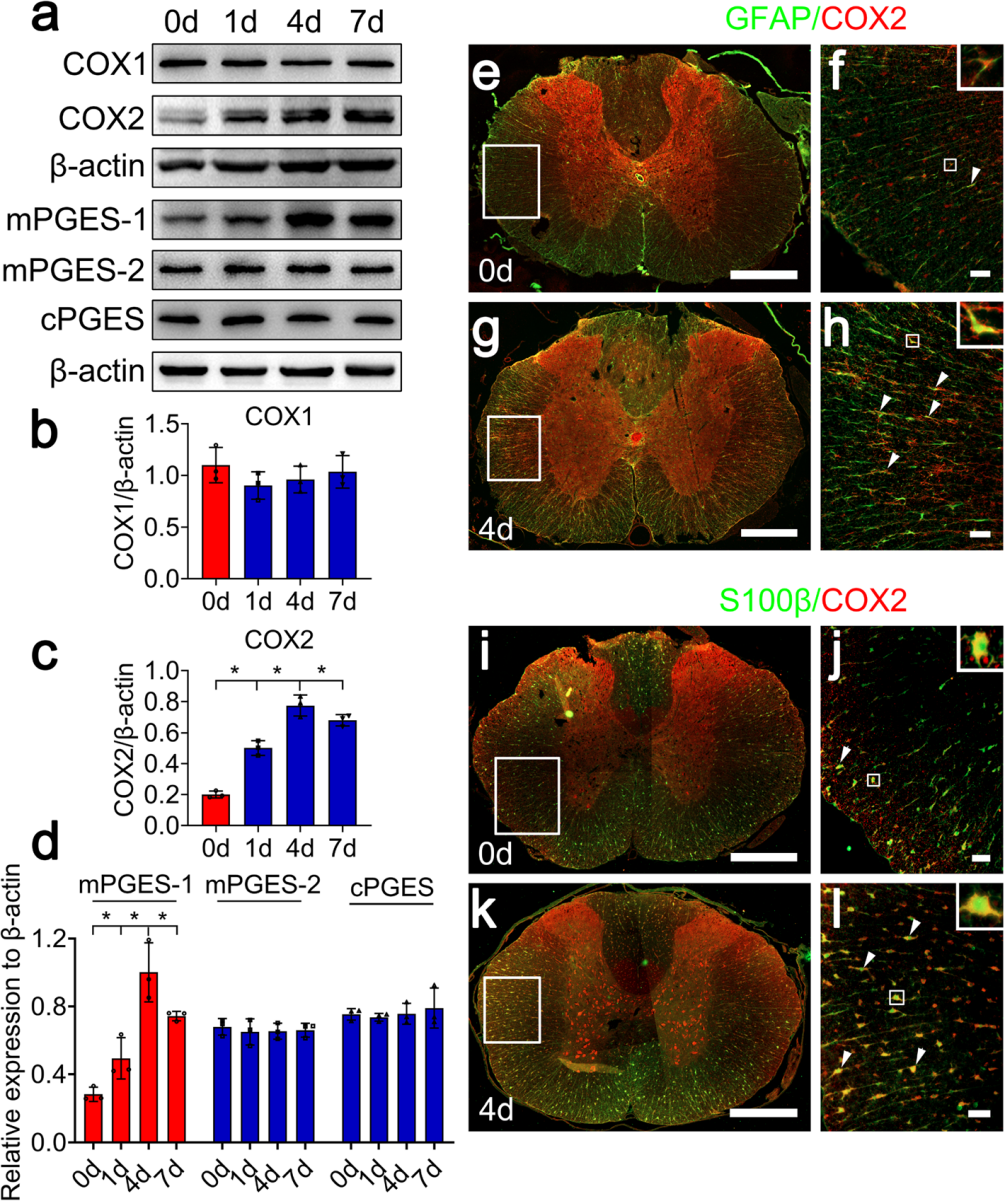
Determination of COX2 protein levels and colocalization of COX2 with astrocytes following SCI. **a** Western blot analysis of COX1, COX2, mPGES-1, mPGES-2, and cPGES at 0, 1, 4, and 7 days following SCI. **b–d** Quantification data as shown in (**a**)**.** Quantities were normalized to endogenous β-actin. **e–l** Immunostaining showed colocalization of COX2 with GFAP- and S100β-positive astrocytes. Rectangle indicates region magnified. Arrowheads indicate the positive signals. Scale bars, 500 μm in (**e**), (**g**), (**i**), and (**k**); 50 μm in (**f**), (**h**), (**j**), and (**l**). Experiments were performed in triplicates. Error bars represent the standard deviation (*P < 0.05)

To elucidate the regulatory correlations between D-DT and COX2 in the astrocytes, we synchronously examined the protein levels of COX1 and COX2, as well as the isoforms of PGE_2_ synthase at the lesion sites following SCI at 0 day, 1 day, 4 days, and 7 days. Results found that the protein levels of COX2 and microsomal PGE synthase-1 (mPGES-1), but not of COX1, mPGES-2, and cytosolic PGE synthase (cPGES), were inducibly expressed with a peak at 4 days following SCI (Fig. 3a–d). Immunofluorescence displayed that COX2 colocalized with GFAP- and S100β-positive astrocytes (Fig. 3e–l). The potential effects of D-DT on the neurons were not taken into account due to undetectable expression of CD74 receptor in the spinal neurons (data not shown). The data indicate that injury-induced D-DT expression is possibly associated with COX2 activation of astrocytes following SCI.

### D-DT is able to activate COX2/PGE_2_ pathway in primary astrocytes

**Fig. 4:**
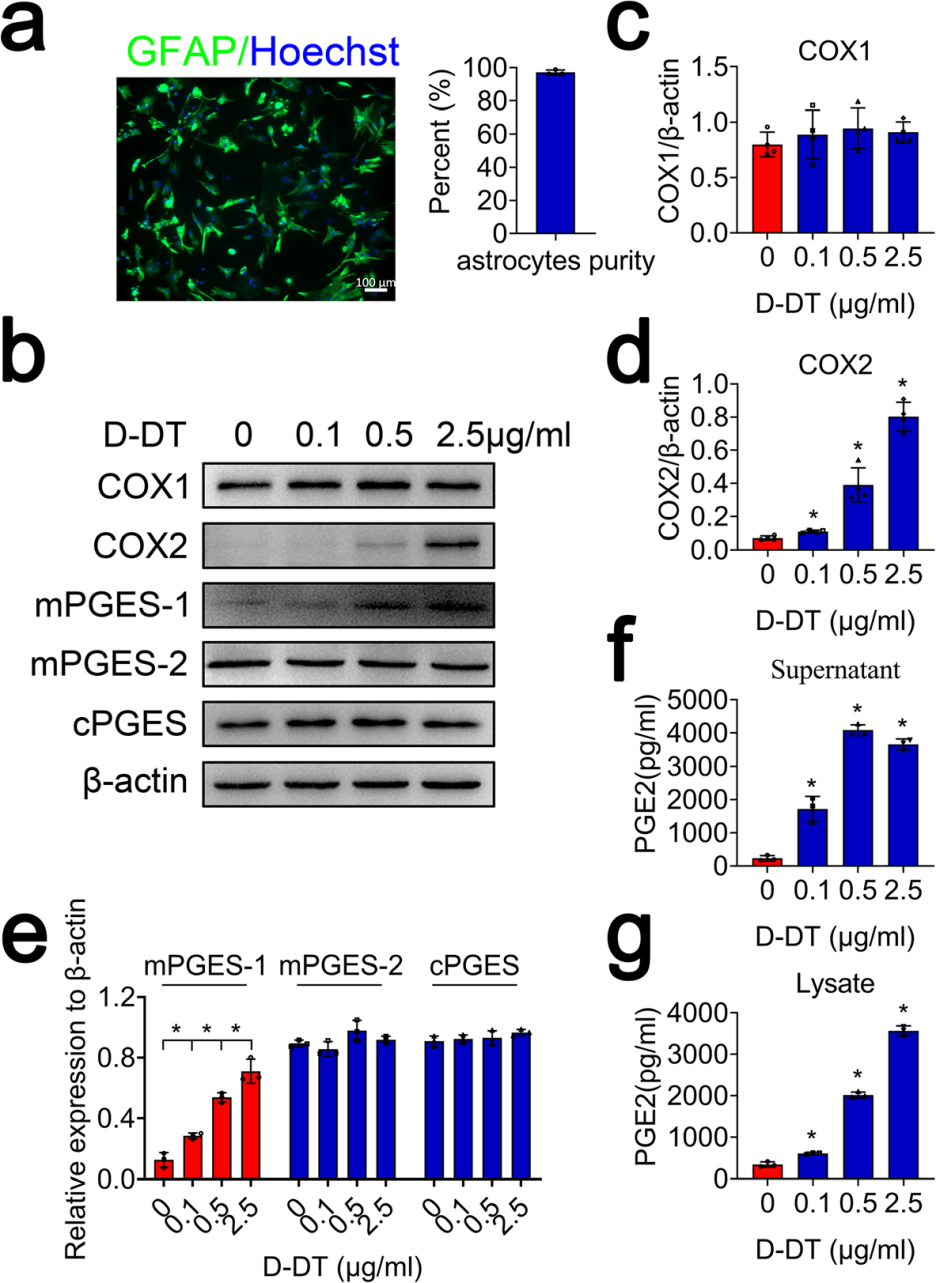
Determination of PGE_2_ synthesis-related protein levels following astrocytes incubation with recombinant D-DT protein. **a** Primary astrocytes stained with GFAP and Hoechst 33342 with the purity over 95%. **b** Western blot analysis of COX1, COX2, mPGES-1, mPGES-2, and cPGES following astrocyte stimulation with 0, 0.1, 0.5, 2.5 μg/ml recombinant D-DT protein for 24 h. **c–e** Quantification data as shown in (**b**). Quantities were normalized to endogenous β-actin. **f**, **g** ELISA determination of PGE_2_ in supernatant and lysate following astrocytes stimulation with 0, 0.1, 0.5, and 2.5 μg/ml recombinant D-DT protein for 24 h. Scale bars, 100 μm in (**a**). Experiments were performed in triplicates. Error bars represent the standard deviation (*P < 0.05)

**Fig. 5:**
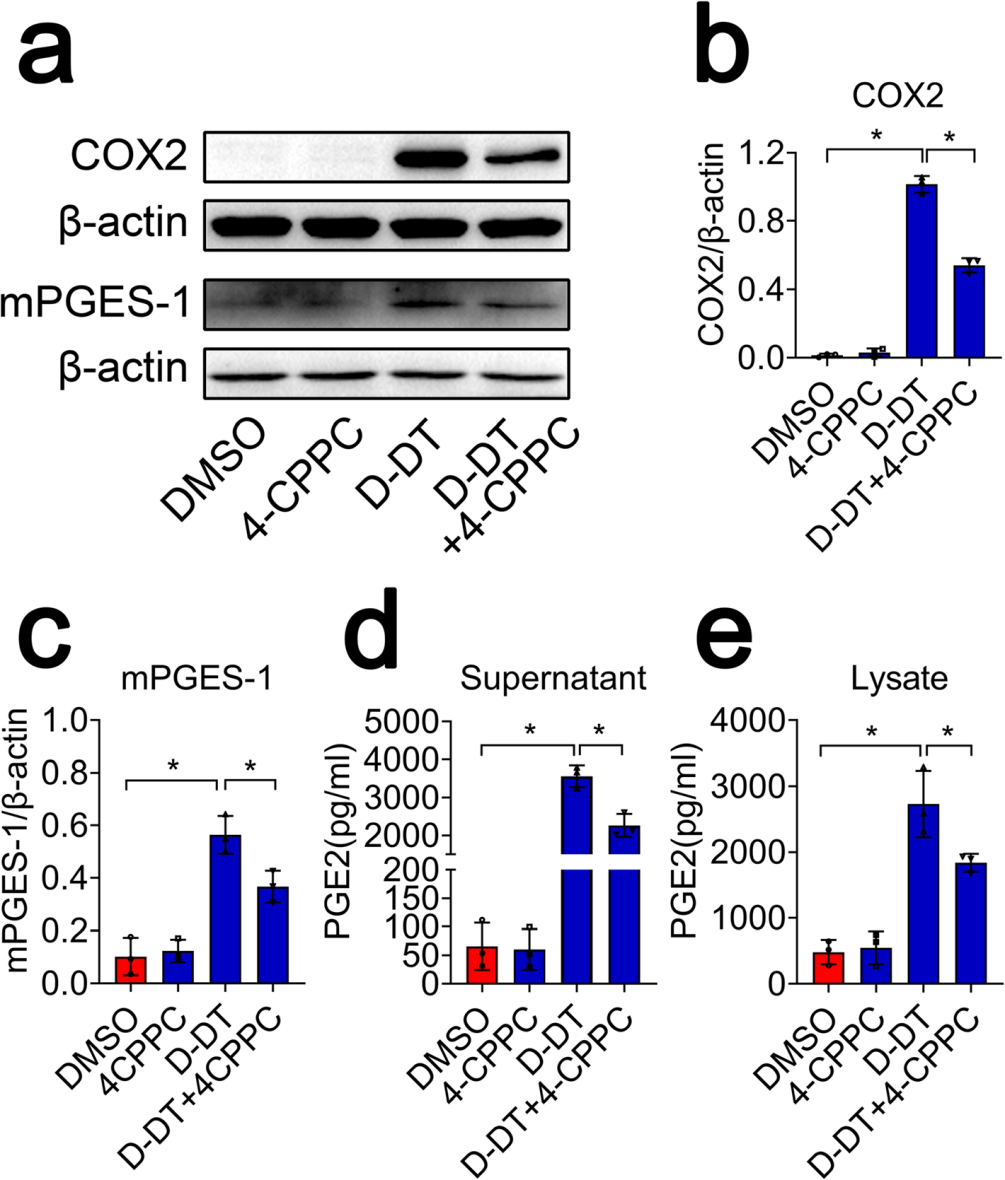
Inhibition of D-DT suppressed production of PGE_2_ from astrocytes. **a** Western blot analysis of COX2 and mPGES-1 following astrocyte stimulation with 1 μg/ml recombinant D-DT in the presence or absence of 100 μM selective inhibitor 4-CPPC for 24 h. **b**, **c** Quantification data as shown in (**a**). Quantities were normalized to endogenous β-actin. **d, e** ELISA determination of PGE_2_ in supernatant and lysate following astrocytes stimulation with 1 μg/ml recombinant D-DT in the presence or absence of 100 μM 4-CPPC for 24 h. Experiments were performed in triplicates. Error bars represent the standard deviation (*P < 0.05)

To unveil the regulatory roles of D-DT on the activation of COX2/PGE_2_ pathway in astrocytes, primary astrocytes were cultured with purity more than 95%, as was evaluated by GFAP staining (Fig. 4a). Astrocytes were stimulated with rat recombinant D-DT protein at concentration of 0–2.5 μg/ml for 24 h. Western blot analysis demonstrated that the protein levels of COX2 and mPGES-1 in the cells were markedly elevated in D-DT dose-dependent manner. However, the expression of COX1, mPGES-2, and cPGES was unaffected (Fig. 4b–e). Meanwhile, the production of PGE_2_ in astrocytes was significantly facilitated by stimulation of D-DT, as shown by ELISA for the culture supernatant and cell lysates (Fig. 4f, g). Addition of 100 μM 4CPPC, a selective inhibitor of D-DT, to the medium containing 1 μg/ml of rat recombinant D-DT, was able to attenuate the effects of D-DT-mediated activation on COX2/PGE_2_ pathway (Fig. 5; Additional file 2). These findings indicate that D-DT is sufficient in activating COX2/PGE_2_ pathway of astrocytes.

### D-DT promotes production of astrocyte PGE_2_ through regulation of COX2

**Fig. 6:**
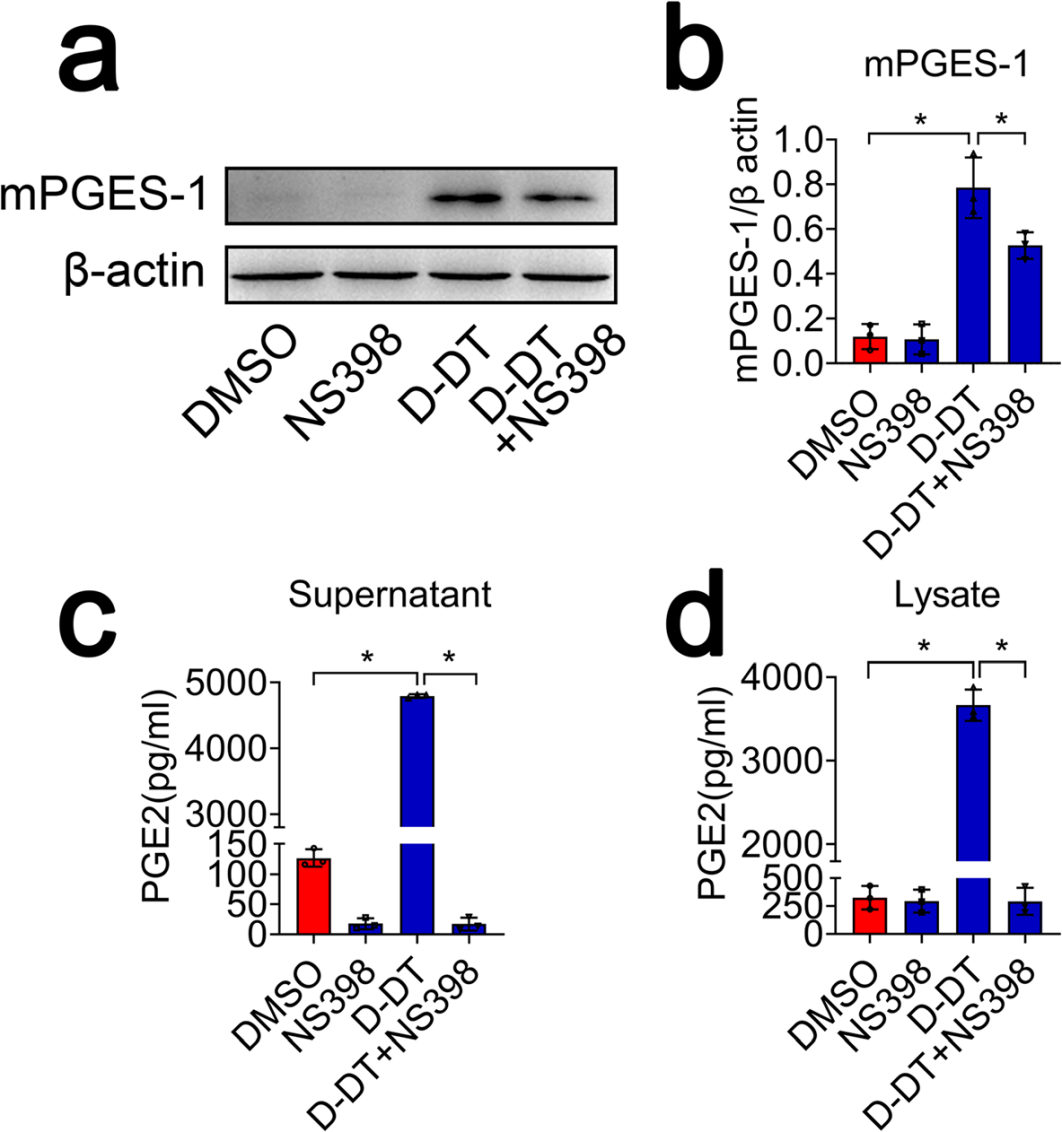
Effects of COX2 selective inhibitor NS398 on the astrocyte production of PGE_2_ in response to D-DT stimulation. **a** Western blot analysis of mPGES-1 following astrocyte treatment with 1 μg/ml recombinant D-DT in the presence or absence of 30 μM NS398 for 24 h. **b** Quantification data as shown in (**a**). Quantities were normalized to endogenous β-actin. **c**, **d** ELISA determination of PGE_2_ in supernatant and lysate following astrocytes stimulation with 1 μg/ml recombinant D-DT in the presence or absence of 30 μM NS398 for 24 h. Experiments were performed in triplicates. Error bars represent the standard deviation (*P < 0.05)

To ascertain whether D-DT-induced production of astrocyte PGE_2_ is under regulation of COX2, astrocytes were preincubated with 30 μM NS398, a selective inhibitor of COX2 for 2 h, prior to stimulation with 1 μg/ml D-DT protein for 24 h. Results showed that incubation of NS398 was able to decrease the protein levels of mPGES-1, as well as the production of PGE_2_ in astrocytes (Fig. 6a–d), while the expression of mPGES-2 and cPGES was unchanged (Additional file 2). These findings indicate that D-DT promotes production of astrocyte PGE_2_ through regulation of COX2.

### D-DT activates COX2/PGE_2_ pathway in astrocytes through interaction with CD74 receptor

**Fig. 7:**
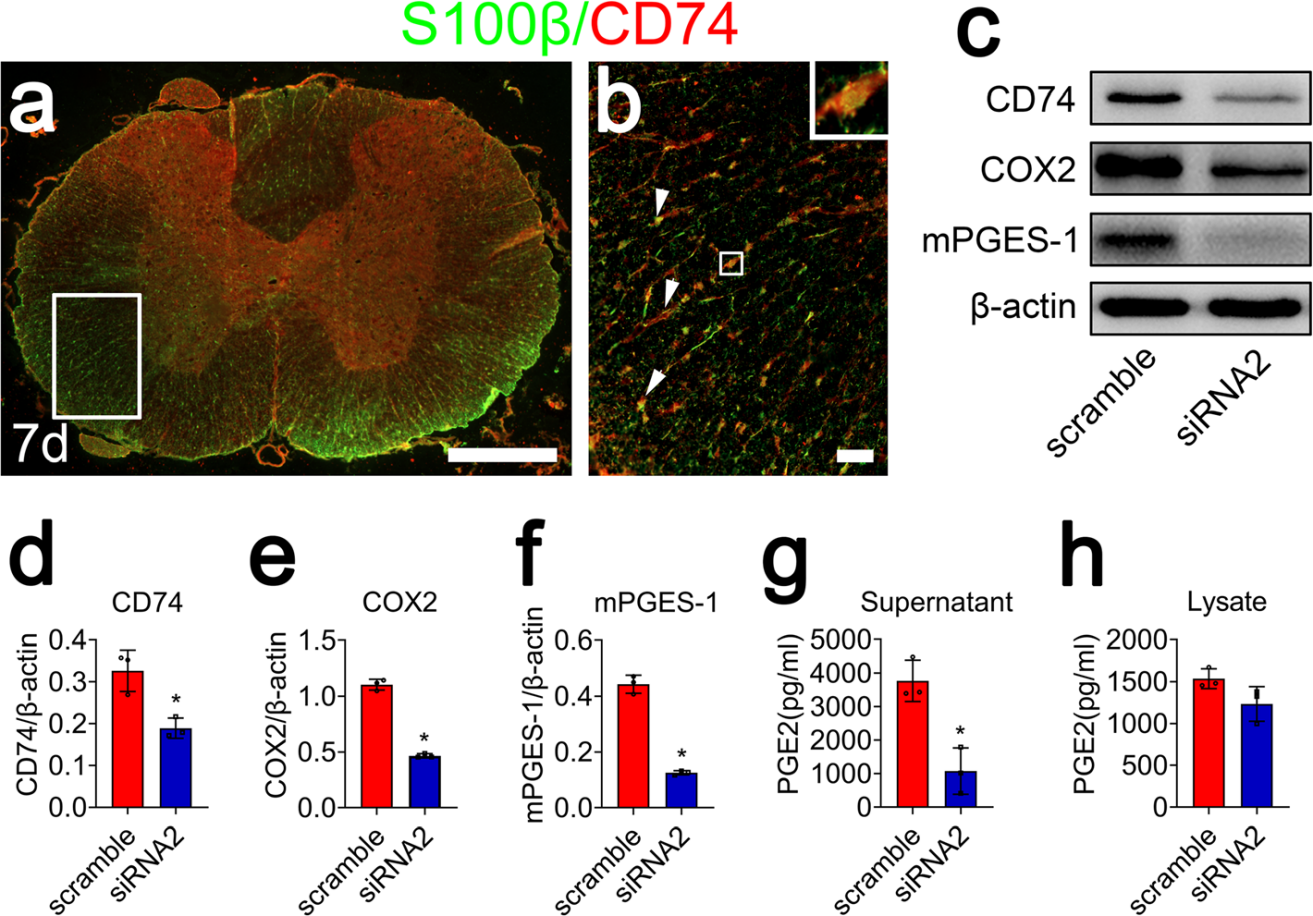
Interference of CD74 receptor decreased D-DT-induced astrocyte production of PGE_2_. **a–b** Immunofluorescence showed colocalization of CD74 with S100β-positive astrocytes in the spinal cord. Rectangle indicates region magnified. Arrowheads indicate the positive signals. **c** Western blot analysis of CD74, COX2, and mPGES-1 following siRNA2 knockdown of CD74 receptor for 48 h, prior to stimulation with 1 μg/ml recombinant D-DT protein for 24 h. **d–f** Quantification data as shown in (**c**). Quantities were normalized to endogenous β-actin. **g**, **h** ELISA determination of PGE_2_ in supernatant and lysate following astrocytes transfected with CD74 siRNA2 for 48 h, followed by treatment with 1 μg/ml recombinant D-DT for 24 h. Experiments were performed in triplicates. Error bars represent the standard deviation (*P < 0.05)

As D-DT protein lacks a pseudo (E)LR domain essential for binding with CXCR2 coreceptor, it implies that D-DT is a more specific ligand for CD74 [30]. To validate the presence of D-DT/CD74 couple in the astrocytes, immunofluorescence was performed to detect the expression of CD74 receptor in astrocytes. Results demonstrated that the membrane receptor colocalized with S100β-positive astrocytes following SCI (Fig. 7a, b). To clarify the intracellular activation of COX2/PGE_2_ pathway was attributed to the D-DT binding with CD74 receptor, we knocked down the expression of the CD74 receptor using siRNA oligonucleotides, and siRNA2 with nearly 50% knockdown efficiency was chosen for the next experiments (Fig. 7c, d). Astrocytes were transfected with CD74 siRNA2 for 48 h, prior to stimulation with 1 μg/ml D-DT for 24 h. The protein levels of COX2 and mPGES-1 (Fig. 7c, e, f), but not of mPGES-2 and cPGES, were significantly reduced (Additional file 2). Accordingly, the production of PGE_2_ in astrocytes markedly decreased following interference of CD74 (Fig. 7g, h). These results indicate that D-DT activates COX2/PGE_2_ signaling pathway through interaction with CD74 receptor.

### D-DT regulates COX2/PGE_2_ pathway through activation of MAPKs

**Fig. 8:**
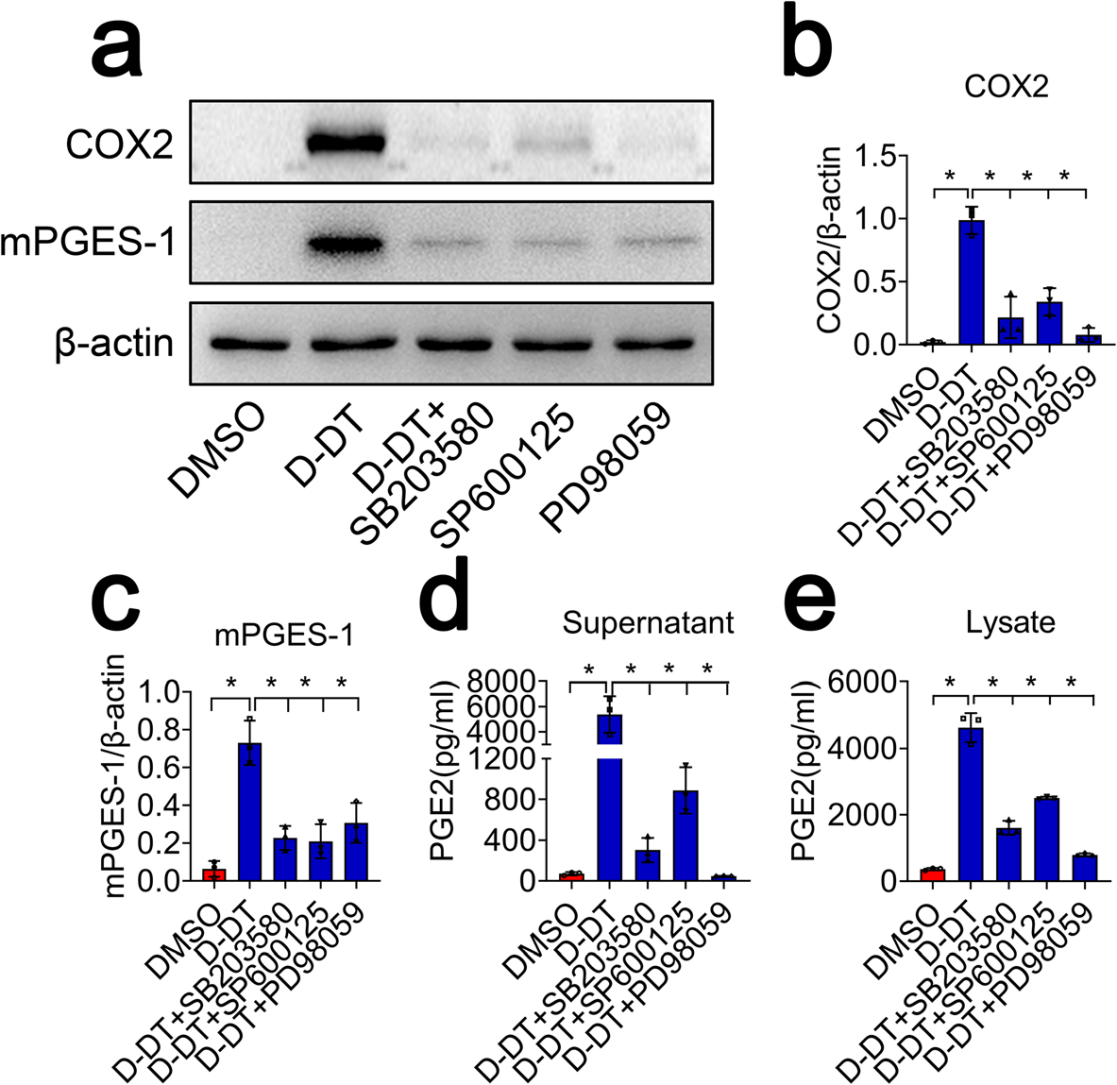
Inhibition of MAPKs inactivated COX2/PGE_2_ pathway of astrocytes. **a** Western blot analysis of COX2 and mPGES-1 following treatment with 1 μg/ml recombinant D-DT in the presence of 10 μM P38 (SB203580), 10 μM JNK (SP600125), or 10 μM ERK (PD98059) inhibitor for 24 h. **b**, **c** Quantification data as shown in (**a**). Quantities were normalized to endogenous β-actin. **d**, **e** ELISA determination of PGE_2_ in supernatant and lysate following astrocytes treatment with 1 μg/ml recombinant D-DT in the presence of 10 μM P38 (SB203580), 10 μM JNK (SP600125), or 10 μM ERK (PD98059) inhibitor for 24 h. Experiments were performed in triplicates. Error bars represent the standard deviation (*P < 0.05)

D-DT has been shown to activate inflammatory responses of macrophages through mediating phosphorylation of ERK1/2, and a costimulation with MIF achieves synergetic effects [30]. To address whether D-DT-mediated COX2/PGE_2_ pathway is under regulation of MAPKs, astrocytes were treated with 10 μM inhibitor of P38 (SB203580), JNK (SP600125), or ERK (PD98059) for 6 h, followed by stimulation with 1 μg/ml recombinant D-DT protein for 24 h. Results demonstrated that the expression of COX2 and mPGES-1 protein, as well as production of PGE_2_ was markedly attenuated following addition of the inhibitor (Fig. 8a–e). However, protein levels of COX1, mPGES-2, and cPGES showed no obvious changes (Additional file 2). These results indicate that D-DT-mediated activation of MAPKs is essential for the regulation of COX2/PGE_2_ pathway.

### 4-IPP is more efficient in blocking COX2/PGE_2_ pathway of astrocytes co-stimulated by MIF and D-DT than 4-CPPC inhibitor

**Fig. 9:**
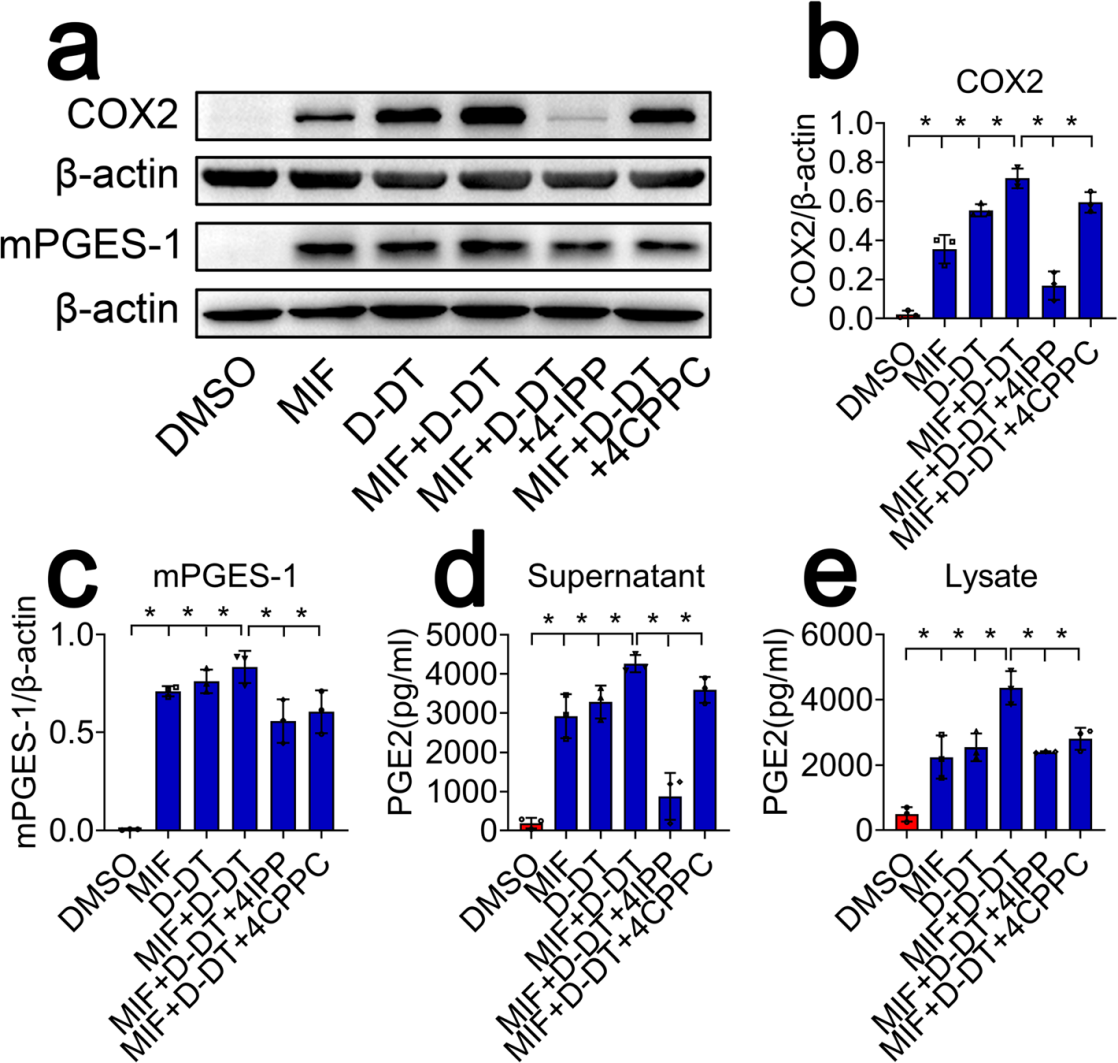
Effects of selective inhibitor on the COX2/PGE_2_ pathway of astrocytes in response to co-stimulation of MIF and D-DT. **a** Western blot analysis of COX2 and mPGES-1 following astrocyte co-stimulation with 1 μg/ml recombinant MIF and/or equivalent D-DT in the presence or absence of 100 μM 4-IPP or 4-CPPC for 24 h. **b**, **c** Quantification data as shown in (**a**). Quantities were normalized to endogenous β-actin. **d**, **e** ELISA determination of PGE_2_ in supernatant and lysate following astrocyte co-stimulation with 1 μg/ml recombinant MIF and/or equivalent D-DT in the presence or absence of 100 μM 4-IPP or 4-CPPC for 24 h. Experiments were performed in triplicates. Error bars represent the standard deviation (*P < 0.05)

**Fig. 10:**
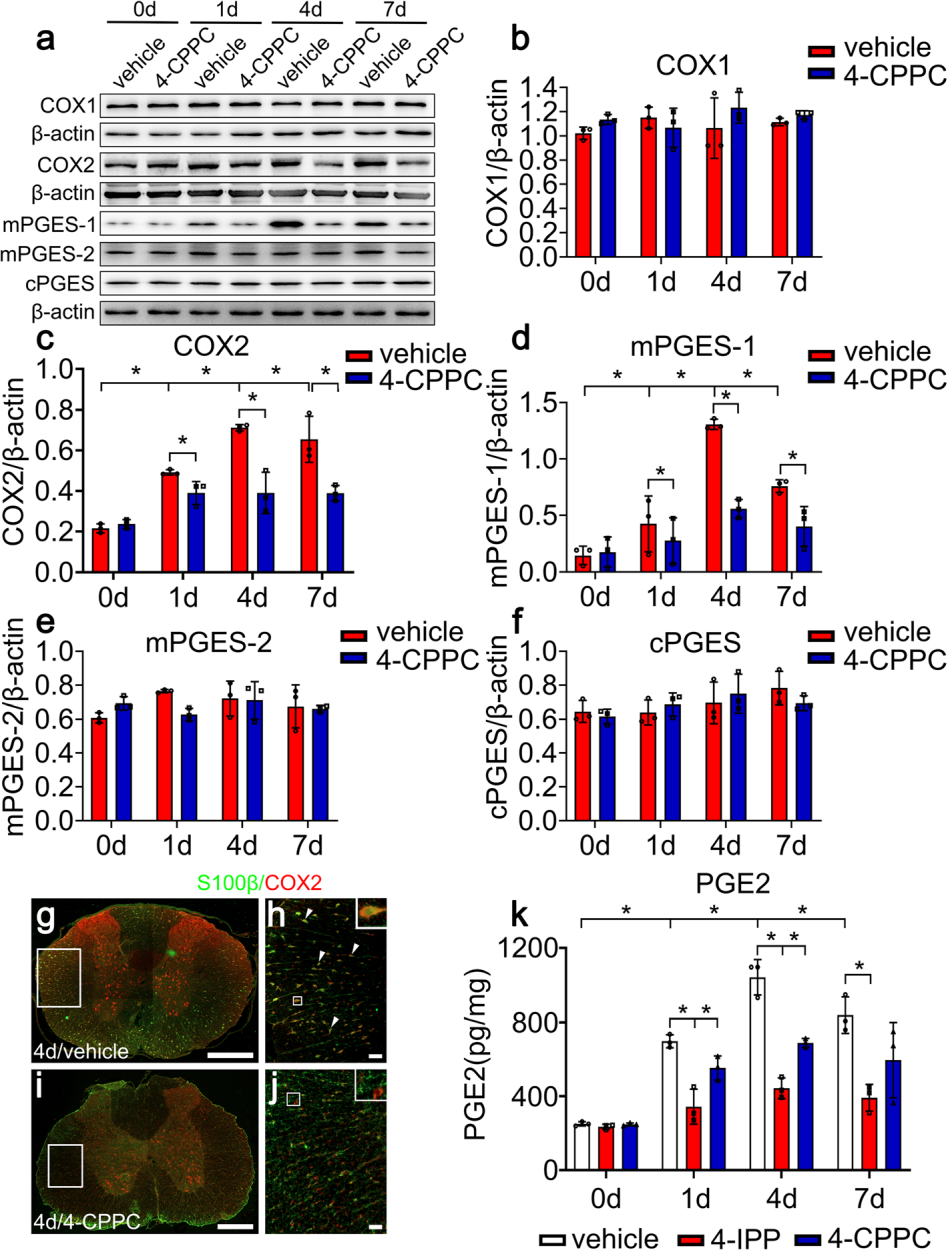
Effects of D-DT inhibition on the production of PGE_2_ following rat SCI. **a** Western blot analysis of COX1, COX2, mPGES-1, mPGES-2, and cPGES at 0, 1, 4, and 7 days following injection of 8 μl of 100 mM 4-CPPC inhibitor at lesion sites of the contused cord. **b–f** Quantification data as shown in (**a**). Quantities were normalized to endogenous β-actin. **g–j** Immunostaining of COX2 in the astrocytes following cord treatment with vehicle (**g**, **h**) or 4-CPPC inhibitor (**i**, **j**) at 4 days. Rectangle indicates region magnified. Arrowheads indicate the positive signals. **k** ELISA determination of PGE_2_ following cord treatment with 4-IPP or 4-CPPC inhibitor at 0, 1, 4, and 7 days, respectively. Experiments were performed in triplicates. Error bars represent the standard deviation (*P < 0.05). Scale bars, 500 μm in (**g**, **i**); 50 μm in (**h**) and (**j**)

As a covalent inhibitor, 4-iodo-6-phenylpyrimidine (4-IPP) is able to inhibit the activity of both MIF and D-DT through forming a covalent bond with Pro-1 of the two proteins [39, 40]. Distinctively, 4-CPPC specifically binds on the C-terminal region of D-DT to make inhibitory effects *via* a major conformational change [41]. Given that both MIF and D-DT protein levels were inducibly elevated at lesion sites following rat SCI [26], the inhibitory effects of 4-IPP and 4-CPPC on the COX2/PGE_2_ pathway of astrocytes were thus evaluated following a synergistic stimulation with MIF and D-DT. Results showed that co-stimulation of the cells with 1 μg/ml recombinant MIF and D-DT significantly promoted activation of COX2/PGE_2_ signaling, compared with those stimulated by each mediator (Fig. 9a–e). However, addition of 100 μM 4-IPP or 100 μM 4-CPPC could attenuate such stimulatory effects, with 4-IPP more efficient than 4-CPPC inhibitor (Fig. 9a–e). As was expected, the protein levels of COX1, mPGES-1, and cPGES were unaffected by those inhibitors (Additional file 2). The data indicate that 4-CPPC specifically inhibits D-DT-induced PGE_2_ production in astrocytes. To examine whether inhibition of D-DT can contribute to reducing the production of PGE_2_ following SCI, rats were intrathecally injected with 8 μl of 100 mM 4-CPPC or vehicle at lesion sites of the cord after contusion. Western blot revealed that protein levels of COX2 and mPGES-1, rather than COX1, mPGES-1, and cPGES in the injured spinal cord were markedly attenuated by 4-CPPC in comparison with the vehicle (Fig. 10a–f). Immunofluorescence was further performed to observe COX2 expression changes in the astrocytes at 4 days following 4-CPPC treatment. Results displayed that COX2 abundance in the astrocytes obviously decreased (Fig. 10g–j). ELISA determination demonstrated that the production of PGE_2_ at lesion sites of the cord accordingly reduced after application of 4-CPPC. Comparatively, the 4-IPP inhibitor was more efficient in reducing PGE_2_ production than 4-CPPC (Fig. 10k). These results indicate inhibition of D-DT activity following SCI is able to suppress astrocyte-mediated COX2/PGE_2_ inflammatory pathway.

### Inhibition of D-DT promotes the recovery of motor function following spinal cord injury

**Fig. 11:**
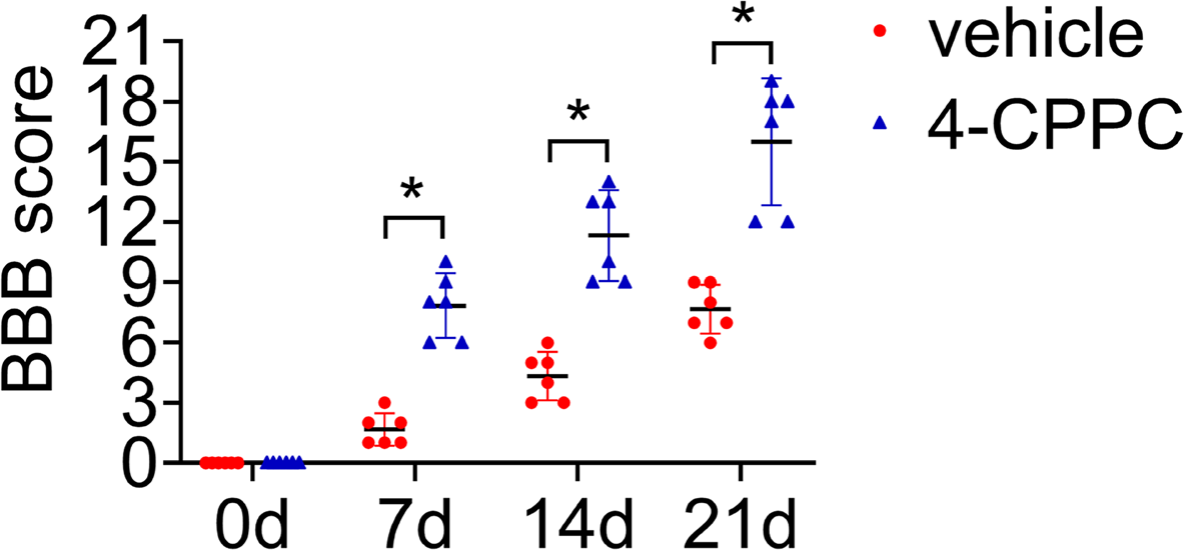
Effects of 4-CPPC on the recovery of motor function following rat SCI. BBB score of hind limb at 0 day, 7 days, 14 days and 21 days following intrathecal injection of 8 μl of 100 mM 4-CPPC or vehicle at the lesion site. Error bars represent the standard deviation (*P < 0.05)

To observe the effect of 4-CPPC on motor function, 8 μl of 100 mM vehicle or 4-CPPC was intrathecally injected at the lesion sites of the cords following contusion. BBB scores were measured during 3 weeks after SCI. Behavioral tests showed that treatment of 4-CPPC significantly improved the recovery of hindlimb locomotor function in comparison with the vehicle (Fig. 11). The data indicate that inhibition of D-DT is beneficial for the recovery of motor function following SCI.

## Discussion

D-DT was firstly identified as an enzyme detectable in the cytoplasm of human melanoma, human liver, and rat organs in 1993, with activity to convert D-dopachrome to 5,6-dihydroxyindolein [42, 43]. Subsequently, the protein was found to distribute in the heart [44], kidney, lung, intestine, and spleen follicular epithelium [30]. D-DT displays high conservation in amino acid sequences across species, implying its functional importance in phylogeny. Interestingly, D-dopachrome is not the physiological substrate of D-DT due to its absence from mammals [28]. This raises a question about the exact physiological significance of the enzyme. Recent years have seen the considerable advances regarding the roles of D-DT, which displays an overlap with those of MIF to some extent [28, 29]. For example, D-DT is distributed in multiple tissues and/or cell types to participate in a broad spectrum of systemic inflammatory diseases including septic shock and arthritis [30, 45, 46]. The protein also plays roles in autoimmune diseases, as well as tumor growth and cell migration in cancer [31, 47]. To date, the expression pattern of D-DT, as well as its cell-specific regulatory functions in the CNS remains unclear. In the present study, we showed that protein levels of D-DT significantly increased following SCI. D-DT was able to induce the production of PGE_2_ from astrocytes in response to cord lesion, which contributed to inflammatory neuropathology as those done by MIF [17]. These suggest that the two family members of MIF still retain a conserved regulatory function as a key player of innate immunity in the CNS.

MIF has been shown to be inducibly expressed within neurons, astrocytes, and microglia of the CNS to mediate neuronal apoptosis and neuroinflammation during neuropathogenesis [25–27, 48]. Deletion of MIF reduces neuronal death and ameliorates functional recovery of the injured spinal cord [25]. Notably, the homolog D-DT was exclusively expressed in the neurons and astrocytes (Figs. 1 and 2), rather than in the microglia, a primary cell source of MIF following SCI. Such difference of cell-specific expression could be partly explained by that injury-induced stimulation is not sufficient in activating D-DT expression in microglia when promoting expression of MIF. The threshold of inflammatory stimuli for D-DT production seems higher than that of MIF, because an equivalent stimulation of LPS in macrophages has been shown to produce 20-fold higher levels of MIF than its homolog [30]. Another alternative mechanism that results in distinct microglia-specific expression of the two cytokines cannot be excluded. D-DT lacks the pseudo (E)LR domain of MIF that is essential for interaction with CXCR2, which is involved in the positive feedback of MIF production [49]. However, the detailed mechanism needs to be elusive in the future. Given that D-DT is not inducibly expressed within microglia, but within astrocytes of the injured spinal cord, microglia might no longer be a promising target in any CNS treatment against D-DT-mediated inflammation.

D-DT was found to promote production of PGE_2_ from astrocytes through the activation of COX2, and the CD74 receptor was required for D-DT action. Similar to MIF, D-DT-mediated activation of MAPKs was involved in the regulation of the COX2/PGE_2_ pathway, suggesting a conserved mechanism for MIF family members in facilitating the production of PGE_2_, even though the structural difference exists between the two proteins [28]. Several evidence has shown that D-DT can perform differential physiological functions with MIF, such as chemotactic activities in recruiting monocytes and leukocytes [30, 50, 51], adipogenesis [36, 52], and wound healing [53]. These functional differences are possibly attributed to the disability of D-DT in binding with CXCR2 [28]. However, CD74 receptor-mediated inflammatory pathway is shared by the two members [30], suggesting evolutionarily functional conservation of both MIF and D-DT protein for binding CD74, and activating downstream inflammatory signaling of multiple cell types.

PGE_2_ is the most important lipid mediator in animals. It plays pathophysiological functions through four receptor subtypes EP1, EP2, EP3, and EP4 [54, 55]. Studies have found that PGE_2_ participates in T helper 1 (Th1)-cell differentiation, Th17-cell expansion, and IL-22 secretion from Th22 cell to induce chronic inflammation and various autoimmune diseases [56–58]. In the mouse model of inflammatory swelling induced by arachidonic acid, PGE_2_ induced by COX activates mast cells through the EP3-Gi/o-Ca^2+^ influx/PI3K pathway to increase vascular permeability and enhance acute inflammation [59]. However, in sepsis, bone marrow stromal cells (BMSCs) produce PGE_2_ to release the anti-inflammatory cytokine IL-10 through EP2 and EP4 receptors on macrophages [60]. Our previous studies have demonstrated that PGE_2_ promotes IL-1β and IL-6, but decreases TNFα expression in macrophages through EP2 receptor to tune inflammatory microenvironment following SCI [17]. In the present study, we showed that D-DT facilitated production of PGE_2_ from astrocytes, which might in turn act similar inflammatory roles in the injured spinal cord.

## Conclusions

Protein levels of D-DT were inducibly elevated in the neurons and astrocytes following SCI, which in turn activated COX2/PGE_2_ signal pathway through regulation of MAPKs. Inhibition of D-DT activity was able to attenuate PGE_2_ production at lesion sites, which is beneficial for the functional recovery of the injured spinal cord.

## Supplementary Information

**Additional file 1: Figure S1.** Colocalization of D-DT with OX42-positive microglia and MBP-positive oligodendrocyte following spinal cord injury at 0d, 1d, 4d, and 7d. Rectangle indicates region magnified. Scale bars, 500 μm in (a), (c), (e), (g), (i), (k), (m), and (o); 50 μm in (b), (d), (f), (h), (j), (l), (n), and (p).

**Additional file 2: Figure S2.** Determination of COX1, mPGES-2 and cPGES protein levels following astrocyte treatment with various inhibitors or knockdown of CD74 expression. **(a)** Western blot analysis of COX1, mPGES-2 and cPGES following astrocytes stimulation with 1 μg/ml recombinant D-DT in the presence or absence of 100 μM selective inhibitor 4-CPPC for 24 h. Quantities were normalized to endogenous β-actin as shown in Fig. 5a. **(b)** Western blot analysis of COX1, mPGES-2 and cPGES following astrocyte treatment with 1 μg/ml recombinant D-DT in the presence or absence of 30 μM NS398 for 24 h. Quantities were normalized to endogenous β-actin as shown in Fig. 6a. **(c)** Western blot analysis of mPGES-2 and cPGES following siRNA2 knockdown of CD74 receptor for 48 h, prior to stimulation with 1 μg/ml recombinant D-DT protein for 24 h. Quantities were normalized to endogenous β-actin as shown in Fig. 7c. **(d)** Western blot analysis of COX1, mPGES-2 and cPGES following treatment with 1 μg/ml recombinant D-DT in the presence of 10 μM P38 (SB203580), 10 μM JNK (SP600125), or 10 μM ERK (PD98059) inhibitor for 24 h. Quantities were normalized to endogenous β-actin as shown in Fig. 8a. **(e)** Western blot analysis of COX1, mPGES-2 and cPGES following astrocyte co-stimulation with 1 μg/ml recombinant MIF and/or equivalent D-DT in the presence or absence of 100 μM 4-IPP or 4-CPPC for 24 h. Quantities were normalized to endogenous β-actin (for COXs or PGE synthase) as shown in Fig. 9a. Experiments were performed in triplicates. Error bars represent the standard deviation (*P < 0.05).

## Data Availability

The datasets used and/or analyzed during the current study are available from the corresponding author on reasonable request.

## Abbreviations

ANOVA: Analysis of variance
CNS: Central nervous system
D-DT: D-dopachrome tautomerase
MIF: Macrophage migration inhibitory factor
DAMP: Damage-associated molecular pattern
PRRs: Pattern recognition receptors
COX: Cyclooxygenases
PGE_2_: Prostaglandin E_2_
mPGES-1: Microsomal PGE synthase-1
PBS: Phosphate buffered saline
MAPK: Mitogen-activated protein kinases
MS: Multiple sclerosis
SCI: Spinal cord injury
GFAP: Glial fibrillary acid protein
MBP: Myelin basic protein
ELISA: Enzyme-linked immunosorbent assay
