# Supplementary material for: D-dopachrome tautomerase activates COX2/PGE2 pathway of astrocytes to mediate inflammation following spinal cord injury
Source: J Neuroinflammation. 2021 Jun 11;18:130. doi: 10.1186/s12974-021-02186-z (PMC8196514; doi:10.1186/s12974-021-02186-z)
Supplement: Supplementary file 1 — Additional file 1: Figure S1. Colocalization of D-DT with OX42-positive microglia and MBP-positive oligodendrocyte following spinal cord injury at 0d, 1d, 4d, and 7d. Rectangle indicates region magnified. Scale bars, 500 μm in (a), (c), (e), (g), (i), (k), (m), and (o); 50 μm in (b), (d), (f), (h), (j), (l), (n), and (p). [file 12974_2021_2186_MOESM1_ESM.pdf]

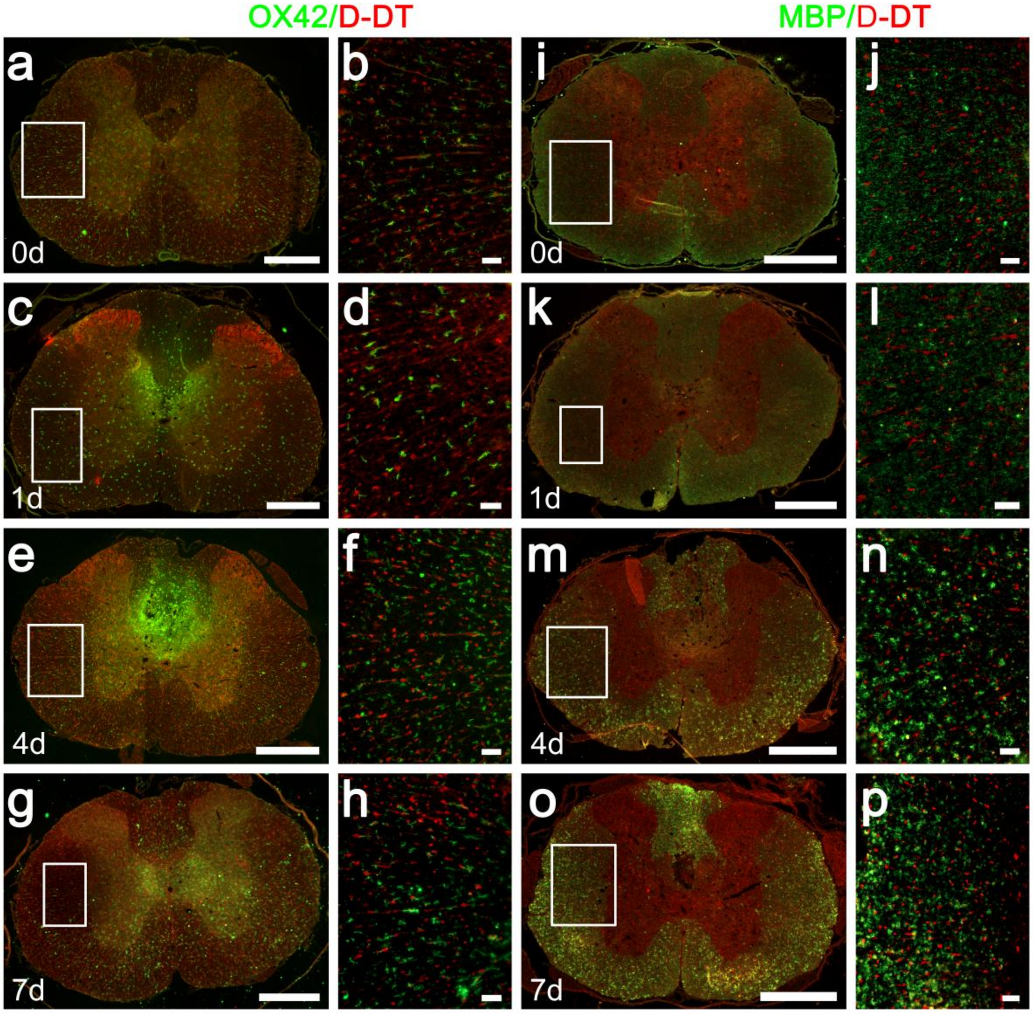

Figure S1. Colocalization of D-DT with OX42-positive microglia and MBP-positive oligodendrocyte following spinal cord injury at 0d, 1d, 4d, and 7d. Rectangle indicates region magnified. Scale bars, 500 μm in (a), (c), (e), (g), (i), (k), (m), and (o); 50 μm in (b), (d), (f), (h), (j), (l), (n), and (p).
