# Supplementary material for: D-dopachrome tautomerase activates COX2/PGE2 pathway of astrocytes to mediate inflammation following spinal cord injury
Source: J Neuroinflammation. 2021 Jun 11;18:130. doi: 10.1186/s12974-021-02186-z (PMC8196514; doi:10.1186/s12974-021-02186-z)
Supplement: Supplementary file 2 — Additional file 2: Figure S2. Determination of COX1, mPGES-2 and cPGES protein levels following astrocyte treatment with various inhibitors or knockdown of CD74 expression. (a) Western blot analysis of COX1, mPGES-2 and cPGES following astrocytes stimulation with 1 μg/ml recombinant D-DT in the presence or absence of 100 μM selective inhibitor 4-CPPC for 24 h. Quantities were normalized to endogenous β-actin as shown in Fig. 5a. (b) Western blot analysis of COX1, mPGES-2 and cPGES following astrocyte treatment with 1 μg/ml recombinant D-DT in the presence or absence of 30 μM NS398 for 24 h. Quantities were normalized to endogenous β-actin as shown in Fig. 6a. (c) Western blot analysis of mPGES-2 and cPGES following siRNA2 knockdown of CD74 receptor for 48 h, prior to stimulation with 1 μg/ml recombinant D-DT protein for 24 h. Quantities were normalized to endogenous β-actin as shown in Fig. 7c. (d) Western blot analysis of COX1, mPGES-2 and cPGES following treatment with 1 μg/ml recombinant D-DT in the presence of 10 μM P38 (SB203580), 10 μM JNK (SP600125), or 10 μM ERK (PD98059) inhibitor for 24 h. Quantities were normalized to endogenous β-actin as shown in Fig. 8a. (e) Western blot analysis of COX1, mPGES-2 and cPGES following astrocyte co-stimulation with 1 μg/ml recombinant MIF and/or equivalent D-DT in the presence or absence of 100 μM 4-IPP or 4-CPPC for 24 h. Quantities were normalized to endogenous β-actin (for COXs or PGE synthase) as shown in Fig. 9a. Experiments were performed in triplicates. Error bars represent the standard deviation (*P < 0.05). [file 12974_2021_2186_MOESM2_ESM.pdf]

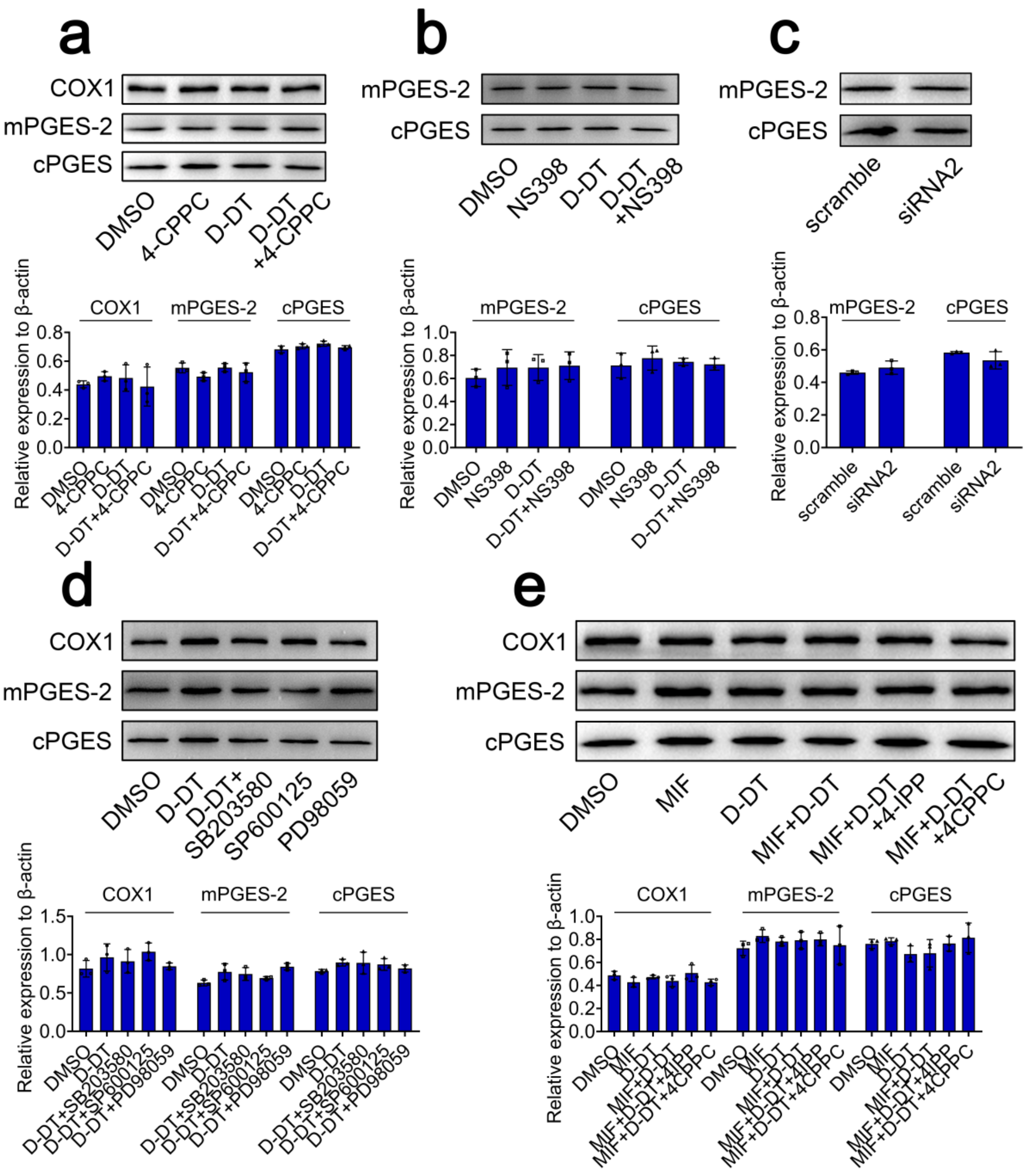


Figure S2. Determination of COX1, mPGES-2 and cPGES protein levels following astrocyte treatment with various inhibitors or knockdown of CD74 expression. **(a)** Western blot analysis of COX1, mPGES-2 and cPGES following astrocytes stimulation with 1 μg/ml recombinant D-DT in the presence or absence of 100 μM selective inhibitor 4-CPPC for 24 h. Quantities were normalized to endogenous β-actin as shown in Fig. 5a. **(b)** Western blot analysis of COX1, mPGES-2 and cPGES following astrocyte treatment with 1 μg/ml recombinant D-DT in the presence

or absence of 30 μM NS398 for 24 h. Quantities were normalized to endogenous β-actin as shown in Fig. 6a. **(c)** Western blot analysis of mPGES-2 and cPGES following siRNA2 knockdown of CD74 receptor for 48 h, prior to stimulation with 1 μg/ml recombinant D-DT protein for 24 h. Quantities were normalized to endogenous β-actin as shown in Fig. 7c. **(d)** Western blot analysis of COX1, mPGES-2 and cPGES following treatment with 1 μg/ml recombinant D-DT in the presence of 10 μM P38 (SB203580), 10 μM JNK (SP600125), or 10 μM ERK (PD98059) inhibitor for 24 h. Quantities were normalized to endogenous β-actin as shown in Fig. 8a. **(e)** Western blot analysis of COX1, mPGES-2 and cPGES following astrocyte co-stimulation with 1 μg/ml recombinant MIF and/or equivalent D-DT in the presence or absence of 100 μM 4-IPP or 4-CPPC for 24 h. Quantities were normalized to endogenous β-actin (for COXs or PGE synthase) as shown in Fig. 9a. Experiments were performed in triplicates. Error bars represent the standard deviation (*$P < 0.05$).
